# *AB*_2_*X*_4_ spinel structures: similarity and differences between the centrosymmetric, *Fd*3*m*, and non-centrosymmetric, *F*4_1_32, space groups

**DOI:** 10.1107/S2052520626004361

**Published:** 2026-05-28

**Authors:** Alla Arakcheeva, Arnaud Magrez, Gervais Chapuis

**Affiliations:** a École Polytechnique Fédérale de Lausanne SB, IPHYS, Crystal Growth Facility Lausanne 1015 Switzerland; b École Polytechnique Fédérale de Lausanne SB, IPHYS, Cubotron Lausanne 1015 Switzerland; Academy of Sciences of the Czech Republic, Czechia

**Keywords:** *AB*_2_*X*_4_ spinel structures, *F*4_1_32 and *Fd*3*m* space groups, lattice complexes, centro- and non-centrosymmetric structures

## Abstract

Many compounds adopting the spinel *AB*_2_*X*_4_ structure are technologically important owing to their tunable physical and chemical properties enabling diverse applications in energy storage, catalysis, magnetism, and functional ceramics. Most of them are traditionally assigned to the centrosymmetric space group *Fd*3*m*. However, the physical properties of some spinels are incompatible with centrosymmetry. This discrepancy is often accounted for by reducing the symmetry to the non-centrosymmetric space group *F*43*m*, allowing thus small atomic displacements from their original position in *Fd*3*m*. In this work, we demonstrate that the loss of the inversion symmetry can occur without any atomic displacements, since the centrosymmetric *Fd*3*m* and non-centrosymmetric *F*4_1_32 space groups are equivalent for structure determination and refinement based on X-ray diffraction data. If consistent with experiment, only the use of an anharmonic model of atomic displacements can distinguish these space groups. This study aims to clarify certain misconceptions regarding the structural symmetry and physical properties of spinel-type compounds.

## Abstract 2

Both centrosymmetric, *Fd*3*m*, and non-centrosymmetric, *F*4_1_32, space groups cannot be distinguished in solving, refining and describing *AB*_2_*X*_4_ spinel structures in the harmonic approximation. They can be distinguished only by using the third-order tensor approximation of anisotropic displacement parameters.

## 1. Introduction

The spinel structure type is adopted by a broad family of *AB*_2_*X*_4_ compounds, many of which exhibit technologically relevant magnetic, electronic, optical, or electrochemical properties. This structural versatility has enabled their use in applications ranging from energy storage and catalysis to magnetic and functional ceramics (Srikala *et al.*, 2024 ▸; Wang *et al.*, 2023 ▸; Rafie *et al.*, 2025 ▸; Arshad *et al.*, 2024 ▸; He *et al.*, 2023 ▸; Song *et al.*, 2023 ▸; Shan *et al.*, 2023 ▸; Xu *et al.*, 2023 ▸; Tsurkan *et al.*, 2021 ▸; Narang & Pubby, 2021 ▸; Szablowski *et al.*, 2025 ▸; and many others). Determining the correct space group symmetry is essential for predicting, understanding and tuning their physical properties. Traditionally, the spinel-type *AB*_2_*X*_4_ compounds are assigned to the centrosymmetric space group *Fd*3*m*. However, doubts have arisen for synthetic MgAl_2_O_4_ where inconsistencies between observed physical properties and centrosymmetric symmetry have been reported (Grimes *et al.*, 1983 ▸). Such doubts primarily concern the presence or absence of an inversion centre.

A confirmation of a non-centrosymmetric symmetry, *F*43*m*, has been published for MgAl_2_O_4_ (Grimes *et al.*, 1983 ▸), and ZnFe_2_O_4_ (Dronova *et al.*, 2022 ▸) among others. Furthermore, two reports refer to the non-centrosymmetric space group *F*4_1_32 for *AB*_2_*X*_4_ compounds. One of these concerns ZnFe_2_O_4_ (Dronova *et al.*, 2022 ▸), where the observed *h*00 reflection condition (*h* ≠ 4*n* with integer *n*) is inconsistent with this space group. The other case involves LiMn_1.5_Ni_0.5_O_4_, in which *A* = Li, *B*_2_ = (Mn_1.5_Ni_0.5_) and *X*_4_ = O_4_ in the general formula *AB*_2_*X*_4_ (Amin *et al.*, 2020 ▸). In that study, the authors proposed a phase transition from *Fd*3*m* to its maximal *translationengleiche* subgroup *F*4_1_32, preserving the same translational characteristics. The *A*, *B* and O atomic positions remain identical in both space groups when, origin choice 1 (with 43*m* at the origin) is adopted for *Fd*3*m.*

In this report, we demonstrate that the space groups *Fd*3*m* and *F*4_1_32 are equivalent for the modelling and refinement of spinel-like *AB*_2_*X*_4_ structures based on conventional X-ray diffraction data. In addition, we demonstrate how a non-centrosymmetric model can be applied and refined by carefully selecting the appropriate experimental X-ray wavelength, and incorporating at least third-order tensor of anisotropic displacement parameters (ADPs) in the structure model.

## 2. Comparison of the space groups *Fd*3*m* and *F*4_1_32 for the *AB*_2_*X*_4_ spinel structures

### 2.1. Structural parameters

**Figure 1:**
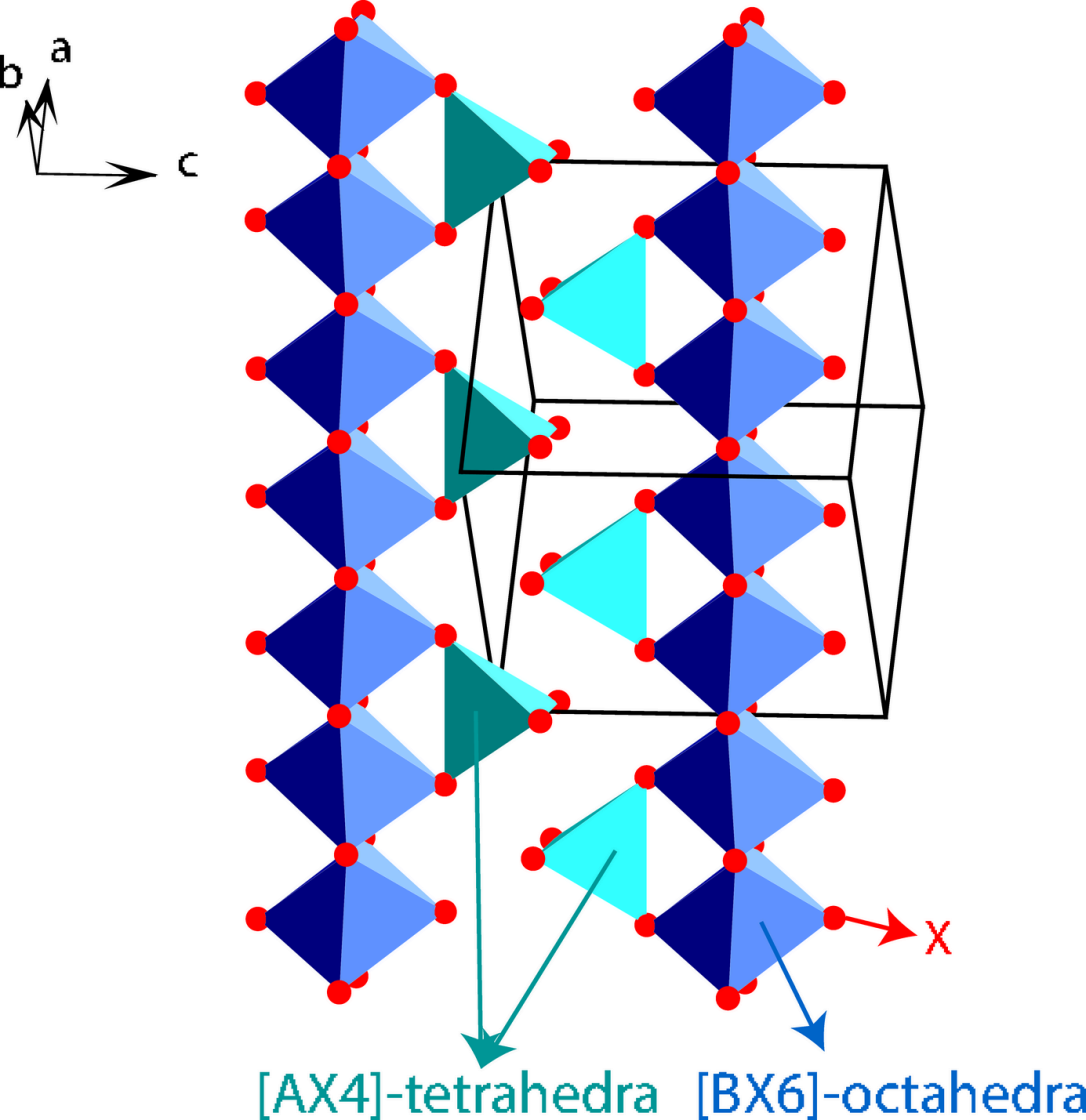
Characteristic fragment of *AB*_2_*X*_4_ spinel-like structure showing its main structure elements. The cubic unit cell is indicated by black lines.

The cubic unit cell of the spinel-like *AB*_2_*X*_4_ structure (Fig. 1 ▸) contains *Z* = 8 formula units: comprising 32 *X* atoms, 8 *A* atoms and 16 *B* atoms. In both space groups *Fd*3*m* and *F*4_1_32, all atoms occupy identical Wyckoff positions with the same multiplicity and atomic coordinates: *X* – 32*e* (*x*, *x*, *x* + symmetry equivalents); *A* – 8*a* (0, 0, 0 + symmetry equivalents); *B* – 16*d* (5/8, 5/8, 5/8 + symmetry equivalents). The complete sets of atomic coordinates for each crystallographic site in both space groups are provided in Table 1 ▸.

Thus, based on structural parameters alone, no distinction can be made between the *Fd*3*m* and *F*4_1_32 space groups. In terms of lattice complexes (Fischer & Koch, 2002 ▸), the crystal structures are also identical in both cases: *A* – 8*a*43*mFd*3*ma* D; *B* – 16*d* .3*mFd*3*mcT*; *X* – 32*e* .3*mFd*3*me* ..2*D*4*xxx*.

### 2.2. Reflection conditions

The general reflection conditions differ slightly between *Fd*3*m* and *F*4_1_32. Specifically, the condition *k* + *l* = 4*n* (where *n* is an integer) is characteristic of *Fd*3*m* but not of *F*4_1_32 (as indicated in Table 1 ▸). However, in *F*4_1_32, additional reflection conditions associated with the *A*, *B* and *X* atomic sites, also satisfying *k* + *l* = 4*n*, effectively suppress this distinction.

Therefore, based solely on the reflection conditions (Table 1 ▸), it is not possible to distinguish between the *Fd*3*m* and *F*4_1_32 space groups for the *AB*_2_*X*_4_ spinel structure.

### 2.3. Resonance scattering and generalized Debye–Waller factor

In general terms, the classical approach to distinguish between centro- and non-centrosymmetric space groups is to take advantage of the resonant scattering effect of X-ray diffraction. In the harmonic approximation, the structure factor can be expressed in the following form:
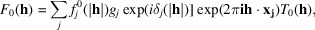
where 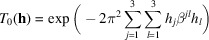
is the Debye–Waller term in the harmonic approximation. Here β*^jl^* is related to *U^jl^* by the equality
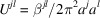
The scattering factor and resonant terms *f*′ and *f*′′ for atom *j* are expressed in the following relations:
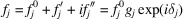
And
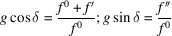
In the presence of resonant scattering, the non-centrosymmetric space group, the relation *I*(**h**) and *I*(−**h**) are different whereas in the centrosymmetric case both intensities are identical. We can calculate the four terms *F*(**h**) and its conjugate F*(**h**), and *F*(−**h**) and its conjugate F*(−**h**) and obtain
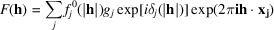

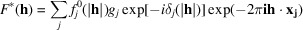

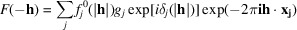

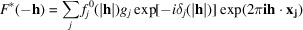


The intensities *I*(**h**) and *I*(−**h**) are proportional to the products *F*(**h**)F*(**h**) and *F*(−**h**)F*(−**h**) respectively. We can see that the products *F*(**h**)F*(**h**) and *F*(−**h**)F*(−**h**) are different by concentrating on the signs of the two exponential terms containing *δ_j_* and **h** · **x**_j_. In the first product *F*(**h**)F*(**h**) the exponential signs of the first term are ++, whereas they are−− in the second term. In the second product *F*(−**h**)F*(−**h**) the exponential signs are +− in the first term and −+ in the second term, which are different from the first product. Consequently, we can conclude that for non-centrosymmetric structures *I*(**h**) and *I*(−**h**) are different.

One may wonder if this difference in intensities could be exploited in our spinel example? Unfortunately, this is not the case. The reason is that in both centro- and non-centrosymmetric cases, each pair of identical atoms *A*, *B* and *X* belong to identical lattice complexes in both space groups. In other words, the two space groups, centrosymmetric and non-centrosymmetric, cannot be distinguished in the *harmonic* approximation of the diffraction model and this is easily confirmed by simulations.

There is, however, some possibility offered by diffraction to distinguish between the centro- and non-centrosymmetric space groups. In the presence of anharmonicity, the Debye–Waller factor can be generalized by including higher-order terms in the power series. If we restrict ourselves to terms up to third-order, we obtain the following generalized expression of the Debye–Waller term (Trueblood *et al.*, 1996 ▸):
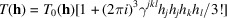
Here γ*^jkl^* are the components of a third-order tensor and for simplification we assume that the summations over the three pairs of identical indices are implied.

**Figure 2:**
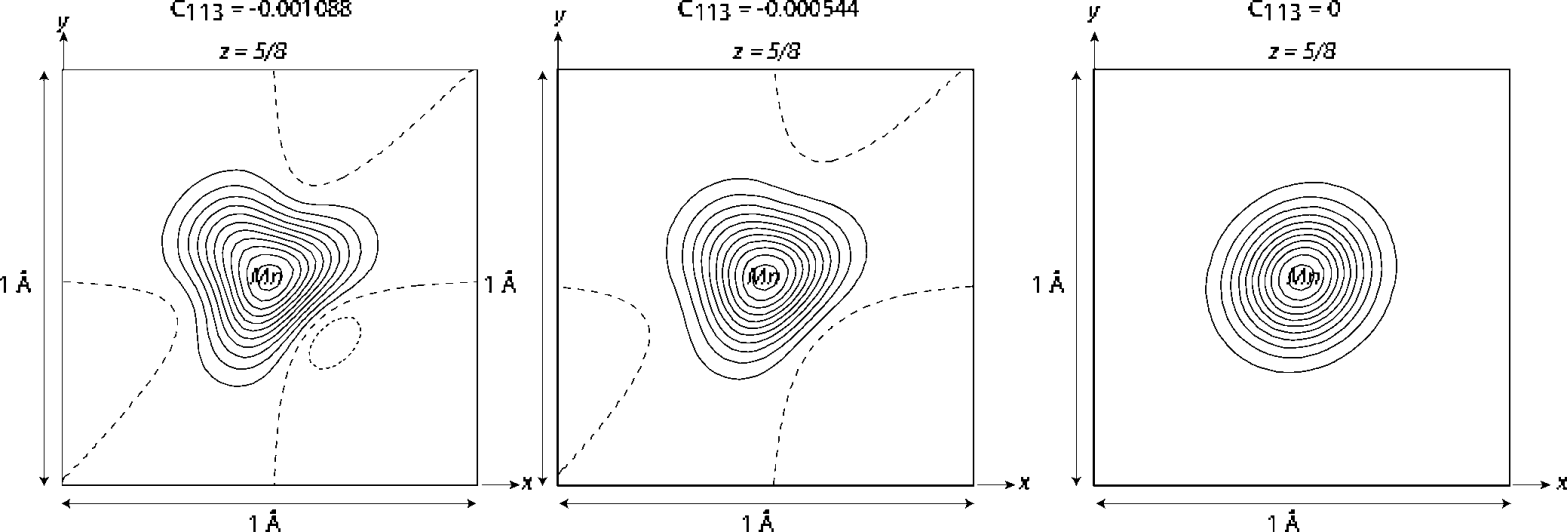
Illustration of the probability distribution function (p.d.f.) obtained using the *C*_113_ term of the third-order ADP tensor for Mn in the simulated spinel structure LiMn_2_O_4_ using Co radiation. The same scale is used for all maps.

Table 2 ▸ shows that the independent ADPs are identical for the *Fd*3*m* and *F*4_1_32 space groups up to second-order tensor approximations for each of *A*, *B*, *X* atom. Using the third-order ADP tensor allows them to be distinguished, provided, of course, that an optimized X-ray wavelength has been selected and that the sensitivity of the X-ray diffraction and the quality of the data are sufficient. The reason concerns the difference in the third-order independent ADP tensor parameters for atoms *X* and *B*: namely, *C*_112_ = *C*_133_ = *C*_223_ ≠ *C*_113_ ≠ 0 in *F*4_1_32, while C_112_ = *C*_133_ = *C*_223_ = *C*_113_ ≠ 0 in *Fd*3*m* for X; *C*_113_ ≠ 0 in *F*4_1_32, while *C*_113_ = 0 in *Fd*3*m* for *B*. Based on the simulation of the LiMo_2_O_4_ structure (see Section 2.4) using Co and Mo radiation (Table 3 ▸), Table 4 ▸ shows the influence of the choice of wavelength, since the *f*′′ value is decisive for F(**h**). For the *B* atom (*B* = Mn in the example), as the heaviest, this value primarily influences F(**h**). Another question arises: is there really an anharmonic shift for the *B* atom? The answer depends on the compound. Fig. 2 ▸ shows the dependence of the probability distribution function (p.d.f.) on the possible anharmonic contribution *C*_113_ of the *B* = Mn atom in the simulated LiMn_2_O_4_ structure. But it is impossible to predict whether the *C*_113_ term for *B* is realistic for a particular *AB*_2_*X*_4_ compound without experimental data.

### 2.4. Example

To simulate the structure of LiMn_2_O_4_ in the centrosymmetric, *Fd*3*m*, and non-centrosymmetric, *F*4_1_32, space groups, the *JANA2006* (Petříček *et al.*, 2014 ▸) software package was used for two radiations, Mo and Co, to highlight the influence of the choice of wavelength on the results. The simulations were performed using ADPs up to the third-order tensor. The same set of atomic coordinates in the same Wyckoff positions was used. The ADPs up to the second-order tensor were fixed at the same values in both space groups. Other details of the structure simulation are given in Table 3 ▸.

Table 4 ▸ summarizes the results of the structure simulations. No difference between squared structure factor |*F*(**h**)|^2^ and |*F*(−**h**)|^2^ can be observed in the centrosymmetric space group *Fd*3*m.* However, in non-centrosymmetric *F*4_1_32 space group, small differences between |*F*(**h**)|^2^ and |*F*(−**h**)|^2^ appear. This difference is higher when Co radiation with anomalous scattering *f*′′(Mn) = 3.555 is applied in comparison to Mo radiation with *f*′′(Mn) = 0.728. This points to the importance of optimizing the wavelength selection if the result needs to discriminate between centrosymmetric and non-centrosymmetric space groups.

## 3. Discussion

The case of LiMn_1.5_Ni_0.5_O_4_ (Amin *et al.*, 2020 ▸) is an interesting one showing the ambiguities resulting from the arbitrary selection of the origins of the specific space groups. This occur frequently while describing series of parent structures in the presence of phase transitions. The selection of the origins of space groups in *International Tables of Crystallography* is based on theoretical considerations which are independent of the behaviour of chemical compounds under considerations.

A convenient way to represent sequences of phase transition is often based on the Bärnighausen tree principle. The tree structure follows a sequence of maximal subgroups of the space groups which are of two types, either *klassen*- or *translationengleich*. We can clearly illustrate our point with the spinel structure LiMn_1.5_Ni_0.5_O_4_.

**Figure 3:**
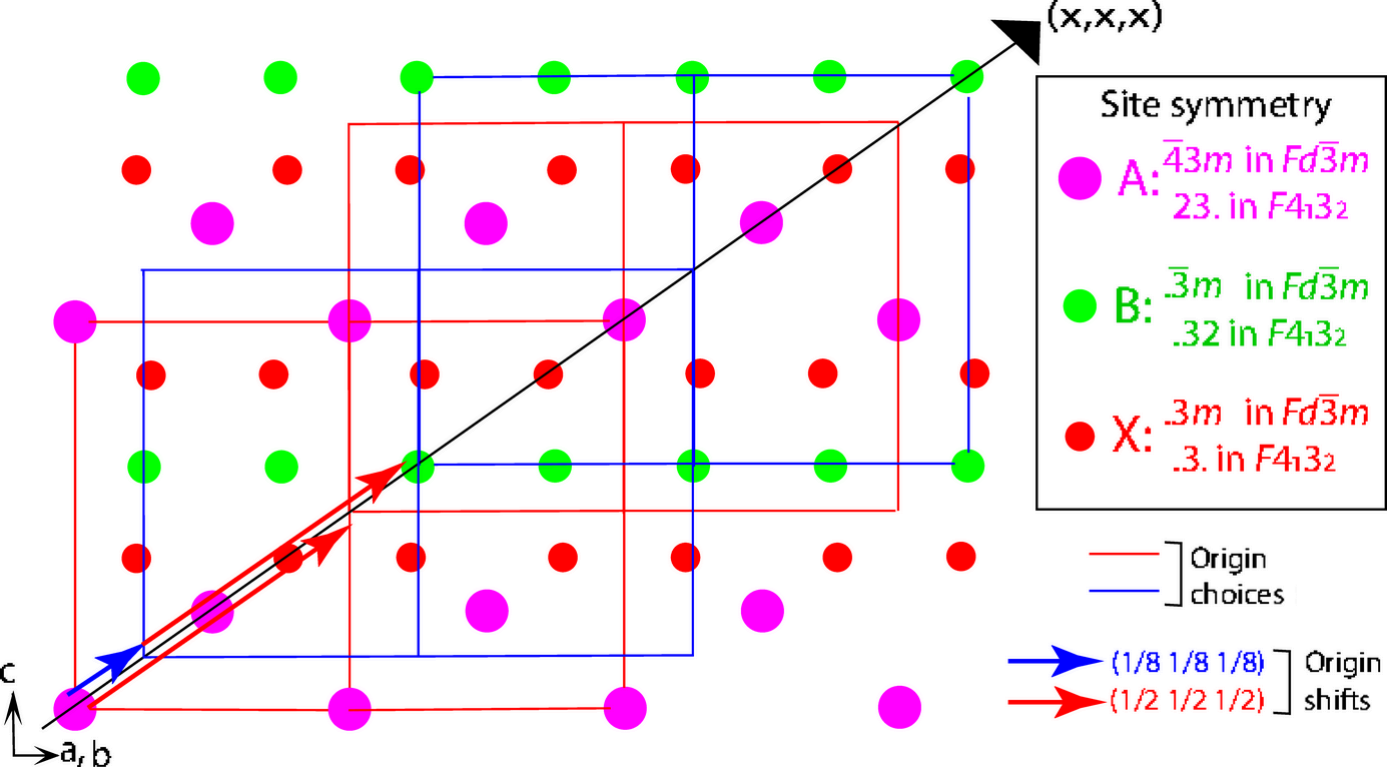
Representative cross-section of the cubic *AB*_2_*X*_4_ spinel structure. Four possible origin choices of the same conventional structure presentation are shown by four projections of the corresponding unit cells along the [110] direction. The *A*, *B*, and *X* independent atoms are located on the indicated threefold axis. Their local symmetry (shown) is independent of the chosen structure representation options; it is systematically lower in *F*4_1_32 compared to *Fd*3*m*. Origin shifts between the specified cells are indicated by arrows. Additional characteristics of the choice of coordinate origin and corresponding sets of atoms are given in Table 6 ▸.

The first transformation from *Fd*3*m* (No. 227) to *F*4_1_32 (No. 210) indicates very different atomic coordinates (Table 5 ▸ according to Figure 3 in the mentioned publication) which at a first glance hints to important structural changes. Once we realise that the higher-symmetry structure is described with origin choice 2 (not indicated in the mentioned publication) and that the lower symmetry one is described in an origin setting which is parent to choice 1 in *Fd*3*m* (Table 6 ▸), we find that the two structures are indistinguishable! The spinel structure can be described and presented by four conventional sets of atomic coordinates. Two of them correspond to two choices of origin in *Fd*3*m*: (i) at 43*m* (origin choice 1) and (ii) at 3*m* (origin choice 2). For each origin choice, two sets of atomic coordinates can be chosen; they are shifted by (1/2, 1/2, 1/2). Of course, the atomic coordinates are related to each other for these origins. Table 6 ▸ and Fig. 3 ▸ illustrate the identity of these choices.

In the same Figure 3 of the mentioned publication, the confusion is also present in the reordering transition from space group *F*4_1_32 (No. 210) to *P*4_1_32 with a maximal subgroup of index k4 (k for *klassengleich*). Here again we would expect small shifts in the atomic parameters. In reality the large difference in the atomic coordinates results from the arbitrariness of the position of the origin of *P*4_1_32 described in the *International Tables for Crystallography*. Thus, while comparing different structures, it is always allowed and recommended to shift the origin of some space groups in order to better illustrate the close parenthood between the pair of structures under considerations.

The next important point to discuss is the concept of lattice complexes introduced and described by Fischer & Koch (2002 ▸) in *International Tables of Crystallography*. Identical lattice complexes are rarely (if ever) found in compounds with low symmetry, but they are indeed very important in compounds with high, especially cubic, symmetry, not only in the spinel group compounds but also in the perovskite group compounds. Indeed, the lattice complex of the perovskite-like *ABX*_3_ structures with the centrosymmetric *Pm3m* space group (No. 221) is identical to the lattice complex of the non-centrosymmetric space group *P*432 (No. 207): site *A* – 1*am*3*mPm*3*maP*; site *B* – 1*bm*3*mPm*3*maP*; site *X* – 3*c4*/*mm* .*mcJ*. This means that for perovskite-like compounds, the difference between centrosymmetric and non-centrosymmetric space groups is very difficult to determine based on X-ray experiments alone. The case of perovskite-like compounds is identical to that of spinel.

An additional point for discussion concerns the reflection conditions, which are very important in distinguishing between the *Fd*3*m* and *F*4_1_32 space groups. The reflection condition *k* + *l* = 4*n* for 0*kl* is valid for both groups (see Table 1 ▸) and only for atoms for which harmonic or anharmonic displacements are absent. According to our simulations using Co radiation and the third-order tensor of ADPs for LiMn_2_O_4_ with *F*4_1_32 space group, three independent reflections, namely 024, 046 and 028, are really present with their intensities 2.0, 7.2 and 7.2 respectively. For comparison, the maximal and minimal reflection intensities of other reflections are 135809.0 (for *hkl* = 004) and 32.3 (for *hkl* = 224), respectively. This means that if the third-order tensor of ADPs is meaningful in the experimental data, the difference between space groups *Fd*3*m* and *F*4_1_32 could be observed. However, in practice, this might be difficult.

## 4. Conclusions

For spinel-like *AB*_2_*X*_4_ compounds, the centrosymmetric *Fd*3*m* space group and the non-centrosymmetric *F*4_1_32 space group are difficult to distinguish based solely on the X-ray diffraction data. We have seen that in the harmonic approximation, X-ray resonance scattering effect cannot be used to distinguish spinel compounds where each atom type belongs to the same lattice complex in both centrosymmetric and non-centrosymmetric space groups. A possible method is to use anharmonic Debye–Waller tensor terms up to at least third order. Consequently, the physical properties of a particular compound are often decisive in determining the presence of inversion symmetry. Similar ambiguity can also arise in perovskite-like *ABX*_3_ compounds, which are also of great practical importance. Furthermore, when comparing different structures, it is always allowed and recommended to shift the origin of some space groups in order to better illustrate the close relationship between the pair of structures under consideration.

## Figures and Tables

**Table 1:** Comparison of the *Fd*3*m* and *F*4_1_32 space groups describing the structure of spinel *AB*_2_*X*_4_ Characteristics of space groups are taken from the *International Tables for Crystallography* (2002), Vol. A, *Space-group symmetry*. Asterisks (*) indicate cyclically permuted coordinates *x*,*y*,*z* and indexes *h*,*k*,*l* are also included. The underlined reflection conditions indicate that the general ones in *Fd*3*m* coincide with the extra ones in *F*4_1_32.

**Space group**	***Fd*3*m* (No. 227); origin choice 1 at 43*m***	***F*4_1_32 (No. 210); origin choice at 23.**
General reflection conditions	hkl*:	hkl*:
	*h* + *k* = 2*n*, *h* + *l* = 2*n*, *k* + *l* = 2*n*; 0kl*:	*h* + *k* = 2*n*, *h* + *l* = 2*n*, *k* + *l* = 2*n*; 0kl*:
	*k* + *l* = 4*n*, *k*,*l* = 2*n*;	*k*, *l* = 2*n*;
	hhl*:	hhl*:
	*h* + *l* = 2*n*;	*h* + *l* = 2*n*;
	h00*:	h00*:
	*h* = 4*n*	*h* = 4*n*

**Characteristics of *A* site, 8*a*. Lattice complex: 8*a*43*mFd*3*m a D***		
**Space group**	***Fd*3*m* (No. 227); origin choice 1 at 43*m***	***F*4_1_32 (No. 210); origin choice at 23.**
*x*, *y*, *z* (fixed)	0, 0, 0, (3/4, 1/4, 3/4)*	0, 0, 0, (3/4, 1/4, 3/4)*

**Characteristics of *B* site, 16*d.* Lattice complex: 16*d* .3*mFd*3*mcT***		
**Space group**	***Fd*3*m* (No. 227); origin choice 1 at 43*m***	***F*4_1_32 (No. 210); origin choice at 23.**
*x*, *y*, *z* (fixed)	5/8, 5/8, 5/8, (3/8, 7/8, 1/8)*	5/8, 5/8, 5/8, (3/8, 7/8, 1/8)*
Site symmetry	.3*m*	.32
Extra reflection conditions	hkl*:	hkl*:
	*h* = 2*n* + 1 or *h*, *k*, *l* = 4*n* + 2 or *h*, *k*, *l* = 4*n*	*h* = 2*n* + 1 or *h*, *k*, *l* = 4*n* + 2 or *h*, *k*, *l* = 4*n* (0kl*: *k + l* = 4*n*)

**Characteristics of *X* site, 32*e.* Lattice complex: 32*e* .3*mFd*3*me* ..2*D*4*xxx***		
**Space group**	***Fd*3*m* (No. 227); origin choice 1 at 43*m***	***F*4_1_32 (No. 210); origin choice at 23.**
*x*, *x*, *x* (*x* is variable)	*xxx*, (−*x*, −*x* + ½, *x* + ½)*	*xxx*, (−*x*, −*x* + ½ *x* + ½)*
	−*x* + ¼, −*x* + ¼, −*x* + ¼, (*x* + ¼, −*x* + ¾, *x* + ¾)*	−*x* + ¼, −*x* + ¼, −*x* + ¼, (*x* + ¼, −*x* + ¾, *x* + ¾)*
Site symmetry	.3*m*	.3.
Extra reflection conditions	No extra conditions	0kl*: *k* + *l* = 4*n*
Reflection conditions for *AB*_2_O_4_ spinel structure type	hkl*: *h* + *k* = 2*n*, *h* + *l* = 2*n*, *k* + *l* = 2*n*;	hkl*: *h* + *k* = 2*n*, *h* + *l* = 2*n*, *k* + *l* = 2*n*;
	0kl*:	0kl*:
	*k* + *l* = 4*n*, *k*,*l* = 2*n*;	*k* + *l* = 4*n*, *k*,*l* = 2*n*;
	hhl*:	hhl*:
	*h* + *l* = 2*n*;	*h* + *l* = 2*n*;
	*h*00*:	*h*00*:
	*h* = 4*n*	*h* = 4*n*

**Table 2:** Comparison of ADPs up to the third order of the tensor in the *Fd*3*m* and *F*4_1_32 space groups describing the structure of spinel *AB*_2_*X*_4_ *U*_*jl*_ and *C*_*jkl*_ define the parameters of the harmonic tensor and the third-order ADP tensor, respectively. Independent parameters are indicated in bold

Atom	*Fd* 3 *m*	*F*4_1_32
*A*	***U*_11_** = *U*_22_ = *U*_33_ ≠ 0	***U*_11_** = *U*_22_ = *U*_33_ ≠ 0
	***U*_12_** = *U*_13_ = *U*_23_ = 0	***U*_12_** = *U*_13_ = *U*_23_ = 0
	***C*_111_** = *C*_112_ = *C*_113_ = *C*_122_ = *C*_222_ = *C*_133_ = *C*_223_ = *C*_233_ = *C*_333_ = 0	***C*_111_** = *C*_112_ = *C*_113_ = *C*_122_ = *C*_222_ = *C*_133_ = *C*_223_ = *C*_233_ = *C*_333_ = 0
	***C*_123_** ≠ 0	***C*_123_** ≠ 0
*B*	***U*_11_** = *U*_22_ = *U*_33_ ≠ 0	***U*_11_** = *U*_22_ = *U*_33_ ≠ 0
	***U*_12_** = *U*_13_ = *U*_23_ ≠ 0	***U*_12_** = *U*_13_ = *U*_23_ ≠ 0
	***C*_111_** = *C*_222_ = *C*_333_ = *C*_123_ = *C*_113_ = *C*_112_ = *C*_122_ = *C*_133_ = *C*_223_ = *C*_233_ = 0	***C*_111_** = *C*_222_ = *C*_333_ = *C*_123_ = 0
		***C*_113_** = *C*_122_ = *C*_233_ = −*C*_112_ = −*C*_133_ = −*C*_223_ ≠ 0
*X*	***U*_11_** = U_22_ = *U*_33_ ≠ 0	***U*_11_** = *U*_22_ = *U*_33_ ≠ 0
	***U*_12_** = U_13_ = *U*_23_ ≠ 0	***U*_12_** = *U*_13_ = *U*_23_ ≠ 0
	***C*_111_** = *C*_222_ = *C*_333_ ≠ 0	***C*_111_** = *C*_222_ = *C*_333_ ≠ 0
	***C*_113_** = *C*_112_ = *C*_122_ = *C*_133_ = *C*_233_ = *C*_223_ ≠ 0	***C*_113_** = *C*_233_ = *C*_122_ ≠ 0
	***C*_123_** ≠ 0	***C*_112_** = *C*_133_ = *C*_223_ ≠ 0
		***C*_123_** ≠ 0

**Table 3:** Details of the LiMn_2_O_4_ crystal structure simulation in two space groups, *Fd*3*m* and *F*4_1_32, using Co *K*α radiation (λ = 1.79027 Å) and Mo *K*α radiation (λ = 0.70926 Å) Using the Co radiation: the anomalous dispersion components for Li: *f*′ = 0.001, *f*′′ = 0.001, for Mn: *f*′ = −2.079, *f*′′ = 3.555 and for O: *f*′ = 0.063, *f*′′ = 0.044. Using the Mo radiation: the anomalous dispersion components for Li: *f*′ = 0.000, *f*′′ = 0.000, for Mn: *f*′ = 0.337, *f*′′ = 0.728 and for O: *f*′ = 0.011, *f*′′ = 0.006. Parameters that differ fundamentally between the *Fd*3*m* and *F*4_1_32 space groups are given in bold.

	Co *K*α radiation		Mo *K*α radiation	
Chemical formula	LiMn_2_O_4_	LiMn_2_O_4_	LiMn_2_O_4_	LiMn_2_O_4_
Crystal system, space group	Cubic, *Fd*3*m* (No. 227)	Cubic, *F*4_1_32 (No. 210)	Cubic, *Fd*3*m* (No. 227)	Cubic, *F*4_1_32 (No. 210)
Origin choice	43*m* (choice 1)	.32	43*m* (choice 1)	.32
Shift of origins	000	000	000	000
Temperature (K)	293	293	293	293
*a* (Å)	8.2261 (2)	8.2261 (2)	8.2261 (2)	8.2261 (2)
*V* (Å^3^)	556.65 (2)	556.65 (2)	556.65 (2)	556.65 (2)
*Z*	8	8	8	8
No. of reflections with *I* > 3σ(*I*)	760	760	1428	1428
(sin θ/λ)_max_ (Å^−1^)	0.558	0.558	0.700	0.700
Ranges of *h*, *k*, *l*	*h* = −9/9, *k* = −9/9, *l* = −9/9	*h* = −9 → 9, *k* = −9/9, *l* = −9/9	*h* = −11/11, *k* = −11/11, *l* = −11/11	*h* = −11/11, *k* = −11/11, *l* = −11/11
Atom (Wyckoff site): coordinates	Li (8*a*): 0, 0, 0	Li (8*a*): 0, 0, 0	Li (8*a*): 0, 0, 0	Li (8*a*): 0, 0, 0
	Mn (16*d*): 5/8, 5/8, 5/8	Mn (16*d*): 5/8, 5/8, 5/8	Mn (16*d*): 5/8, 5/8, 5/8	Mn (16*d*): 5/8, 5/8, 5/8
	O (32*e*): 0.3888, 0.3888, 0.3888	O (32*e*): 0.3888, 0.3888, 0.3888	O (32*e*): 0.3888, 0.3888, 0.3888	O (32*e*): 0.3888, 0.3888, 0.3888
ADPs†	Li:	Li:	Li:	Li:
	*U*_11_ = 0.021807,	*U*_11_ = 0.021807,	*U*_11_ = 0.021807,	*U*_11_ = 0.021807,
	*C*_123_ = 0.001834	*C*_123_ = 0.001834	*C*_123_ = 0.001834	*C*_123_ = 0.001834
	Mn:	Mn:	Mn:	Mn:
	*U*_11_ = 0.010834,	*U*_11_ = 0.010834,	*U*_11_ = 0.010834,	*U*_11_ = 0.010834,
	*U*_12_ = 0.00151,	*U*_12_ = 0.00151,	*U*_12_ = 0.00151,	*U*_12_ = 0.00151,
	***C*_113_ = 0**	***C*_113_ = −0.001088**	***C*_113_ = 0**	***C*_113_ = −0.001088**
	O:	O:	O:	O:
	*U*_11_ = 0.015756,	*U*_11_ = 0.015756,	*U*_11_ = 0.015756,	*U*_11_ = 0.015756,
	*U*_12_ = 0.00224,	*U*_12_ = 0.00224,	*U*_12_ = 0.00224,	*U*_12_ = 0.00224,
	*C*_111_ = 0.002712,	*C*_111_ = 0.002712,	*C*_111_ = 0.002712,	*C*_111_ = 0.002712,
	***C*_112_ = 0**,	***C*_112_ = −0.001799**,	***C*_112_ = 0**,	***C*_112_ = −0.001799**,
	*C*_113_ = −0.000956,	*C*_113_ = −0.000109,	***C*_113_** = −0.000956,	*C*_113_ = −0.000109,
	*C*_123_ = −0.000231	*C*_123_ = 0.00089	*C*_123_ = −0.000231	*C*_123_ = 0.00089

† Only independent ADPs are shown.

**Table 4:** Comparison of the squared structure factors amplitudes, |*F*(**h**)|^2^ and |*F*(−**h**)|^2^, for a few representative reflections of the LiMn_2_O_4_ structure simulated in space groups *Fd*3*m* and *F*4_1_32 using Mo and Co radiations and the third-order ADP tensor

	Co radiation		Mo radiation	
	|*F*(**h**)|^2^ and |*F*(−**h**)|^2^	Δ|*F*|^2^ (%)	|*F*(**h**)|^2^ and |*F*(−**h**)|^2^	Δ|*F*|^2^ (%)
(*hkl*)	*Fd* 3 *m*		*Fd* 3 *m*	
(157) and (1 5 7)	3081.2 and 3081.0	0	4364.6 and 4364.6	0
(135) and (1 3 5)	8630.7 and 8630.7	0	11331.8 and 11331.8	0
(246) and (2 4 6)	193.9 and 193.9	0	199.3 and 199.3	0
(248) and (2 4 8)	82.1 and 82.1	0	62.3 and 62.3	0

(*hkl*)	*F*4_1_32		*F*4_1_32	
(157) and (1 5 7)	3105.6 and 3112.9	0.2	3242.6 and 3243.7	0.03
(135) and (1 3 5)	8678.7 and 8686.7	0.09	8396.1 and 8397.2	0.01
(246) and (2 4 6)	222.8 and 180.0	21.2	156.6 and 150.2	4.2
(248) and (2 4 8)	99.7 and 72.6	10.7	51.6 and 47.6	8.1

**Table 5:** Atomic coordinates of Li(Mn,Ni)_2_O_4_ in *Fd*3*m* and *F*4_1_32 space groups after Amin *et al.* (2020 ▸)

Crystal system, space group	Cubic, *Fd*3*m* (No. 227)	Cubic, *F*4_1_32 (No. 210)
Origin choice	Not indicated	Not indicated
Atom (Wyckoff site): coordinates	Li (8*b*): 3/8, 3/8, 3/8	Li (8*a*): 1/2, 1/2, 1/2
	Mn,Ni (16*c*): 0, 0, 0	Mn,Ni (16*d*): 1/8, 1/8, 1/8
	O (32*e*): 0.2368, 0.2368, 0.2368	O (32*e*): 0.3618, 0.3618, 0.3618

**Table 6:** Atomic positions in four possible unit cells characteristic of the same *AB*_2_*X*_4_ cubic spinel structure in both *Fd*3*m* and *F*4_1_32 space groups For better comparison with Fig. 3 ▸, only coordinates of type (*xxx*) located on one of the four axes of threefold symmetry are indicated. Coordinates listed in *International Tables for Crystallography* are given in bold.

	Origin choice 1 at 43*m* in *Fd*3*m*, accordingly at 23. in *F*4_1_32		Origin choice 2 at .3*m* in *Fd*3*m*, accordingly at .32 in *F*4_1_32	
Origin shift	(0, 0, 0)	(1/2, 1/2, 1/2)	(1/8, 1/8, 1/8)	(1/8, 1/8, 1/8) + (1/2, 1/2, 1/2) = (5/8, 5/8, 5/8)
Coordinate shift	(0, 0, 0)	(−1/2, −1/2, −1/2)	(−1/8, −1/8, −1/8)	(−5/8, −5/8, −5/8)
*A*	**8*a*: (0, 0, 0)**;	**8*b*: (1/2, 1/2, 1/2)**;	**8*a*:** (7/8, 7/8, 7/8);	**8*b*: (3/8, 3/8, 3/8);**
	(1/4, 1/4, 1/4)	(3/4, 3/4, 3/4)	**(1/8, 1/8, 1/8)**	(5/8, 5/8, 5/8)
*B*	**16*d*: (5/8, 5/8, 5/8)**	**16*c*: (1/8, 1/8, 1/8)**	**16*d*: (1/2, 1/2, 1/2)**	**16*c*: (0, 0, 0)**
*X*	**32*e***:	**32*e***:	**32*e***:	**32*e***:
	**(*x*_1_, *x*_1_, *x*_1_)**;	(*x*_1_ − ½, *x*_1_ − ½, *x*_1_ − ½);	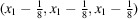 ;	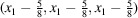 ;
	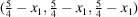 = (¼ − *x*_1_, ¼ − *x*_1_, ¼ − *x*_1_)	(¾ − *x*_1_, ¾ − *x*_1_, ¾ − *x*_1_)	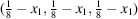	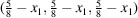
*X* = O in Li(Mn,Ni)_2_O_4_	**32*e*:**	**32*e*:**	**32*e*:**	**32*e*:**
	(0.862, 0.862,0.862);	(0.362, 0.362, 0.362);	(0.737, 0.737, 0.737);	(0.237, 0.237, 0.237);
	(0.388, 0.388, 0.388)	(0.888, 0.888, 0.888)	(0.263, 0.263, 0.263)	(0.763, 0.763, 0.763)
